# Reconfigurable integrated structures with functions of Fabry–Perot antenna and wideband liquid absorber for radar system stealth

**DOI:** 10.1038/s41598-023-41934-4

**Published:** 2023-09-06

**Authors:** Yukun Zou, Xiangkun Kong, Zuwei Cao, Xinyu Zhang, Yongjiu Zhao

**Affiliations:** 1 Key Laboratory of Radar Imaging and Microwave Photonics, Nanjing University of Aeronautics and Astronautics, Nanjing, 210016 People’s Republic of China; 2 State Key Laboratory of Millimeter Waves, Southeast University, Nanjing, 210096 People’s Republic of China

**Keywords:** Applied physics, Electrical and electronic engineering

## Abstract

This paper proposes a functionally reconfigurable integrated structure of a Fabry–Perot (FP) antenna and wideband liquid absorber. First, a two-layer partial reflecting surface (PRS) has been designed. Then, a patch antenna is used to act as the source antenna. By combining the source antenna with the PRS, an FP antenna has been designed. What’s more, taking full advantage of the reflective properties of PRS, a liquid broadband absorber is then designed. Last, the integrated structure with two functions has been realized. It can be used as the FP antenna or the liquid absorber through the extraction and injection of ethanol. In this way, it is effective to switch between stealth and detection states which can be used in different electromagnetic environments. The PRS is elaborately tailored to serve as both a component of the FP antenna and the metal ground of the broadband liquid absorber. Then the integrated structure is realized by combining the FP antenna with the liquid absorber. The PRS is composed of patches on the top layer of the substrate and the square loop on the bottom. The liquid absorber is composed of a 3-D printed container, 45% ethanol layer and the PRS is used to serve as the metal ground. The formula of Mie resonance theory has been extended and used to design the liquid absorber. The gain of the antenna is 19.7 dBi when the ethanol is extracted. When the ethanol is injected, a wideband liquid absorber is achieved. The absorption band (*S*_11_ < − 10 dB) ranges from 4 to 18 GHz. The absorption bandwidth is over 133%. The monostatic RCS reduction bands of the structure with ethanol range from 4 to 18 GHz and the average RCS reduction is 28.4 dBsm. The measured and simulated results are in good agreement.

## Introduction

Fabry–Perot (FP) which is also known as partially reflecting surface (PRS) antennas, 2-D leaky-wave antennas have attracted more and more attention in stealth platforms, sensing systems, and satellite communications because of their simple configurations and high directivity^1–4^. With the development of radar detection technologies, it is important to improve the stealth properties of antennas which play a key role in radar systems in the military background. The RCS is an evaluation criterion of stealth properties. Therefore, it is necessary to reduce the RCS of FP antennas.

Recently, many types of metasurfaces about the RCS reduction of FP antennas have been published. Loading absorbing structures (AS) is an efficacious way to reduce FP antennas RCS. By designing the PRS and the (AS) together^5–8^, the incident waves will be absorbed so that the RCSs of antennas will be reduced. In this way, the antenna's gain often decreases. To maintain the gain of F–P antennas, chessboard-layout metasurfaces are applied to reduce the RCS of FP antennas^9–12^. By locating two kinds of artificial magnetic conductors (AMC) on the top of PRS, the RCS of the antenna will be reduced through the phase cancellation between the incident and reflected waves. Another way to reduce the RCS of the FP antenna is to apply the polarization rotation metasurfaces^13–15^ to the FP antenna. In this way, the co-polarization waves will be transformed into cross-polarization waves so that the RCS of the antenna will be reduced. Coding metasurfaces^16^ have also been applied to the RCS reduction of FP antennas. The incident waves will be scattered in all other directions by encoding techniques. Beyond these, metasurface has been also applied to the antenna systems to broaden the operating frequency band, improve radiation efficiency and gain and suppress the mutual coupling^17–19^.

The technologies proposed above are all based on inactive material. The properties of the antenna keep unchanged once processed. Meanwhile, this can’t adapt to the complicated electromagnetic environment. With the design of active electronic components, such as p-i-n diodes^20,21^, varactors^22,23^ and RF switches^24^, many multifunctional antennas have been designed which can adapt to complex electromagnetic environments. Unfortunately, these active electromagnetic components often affect the performance of antennas because of the parasitic effect and complex feed networks. So far, many liquid materials have been applied to absorbers^25–28^, antennas^29,30^, frequency selective surfaces^31,32^ and so on because of their wide absorption band, transparency, reconfigurable properties, low-RCS, and low price.

To overcome the challenges of reconfigurable properties of FP antennas. This paper proposes a functionally reconfigurable integrated structure of FP antenna and wideband liquid absorber. The novelties and contributions of the design can be summarized as follows: (1) The liquid absorber and PRS of the FP antenna are designed integrally. Under the premise of not affecting the high gain of the antenna, the function switching of the wideband RCS reduction absorber is realized. (2) The formula of the Mie resonance theory has been extended and used to design different shapes of liquid absorbers. (3) Under the same height and metal ground condition, the topology of the wideband liquid absorber is constructed and selected.

This paper will go as follows. First, the working mechanism of the integrated structure has been introduced. Then, the permittivity of the pure water and 45% ethanol solution is discussed. What’s more, the design process of the liquid absorber and FP antenna has been discussed. Last, the property of the integrated structure has been introduced.

### Working mechanism of the integrated structure

**Figure 1:**
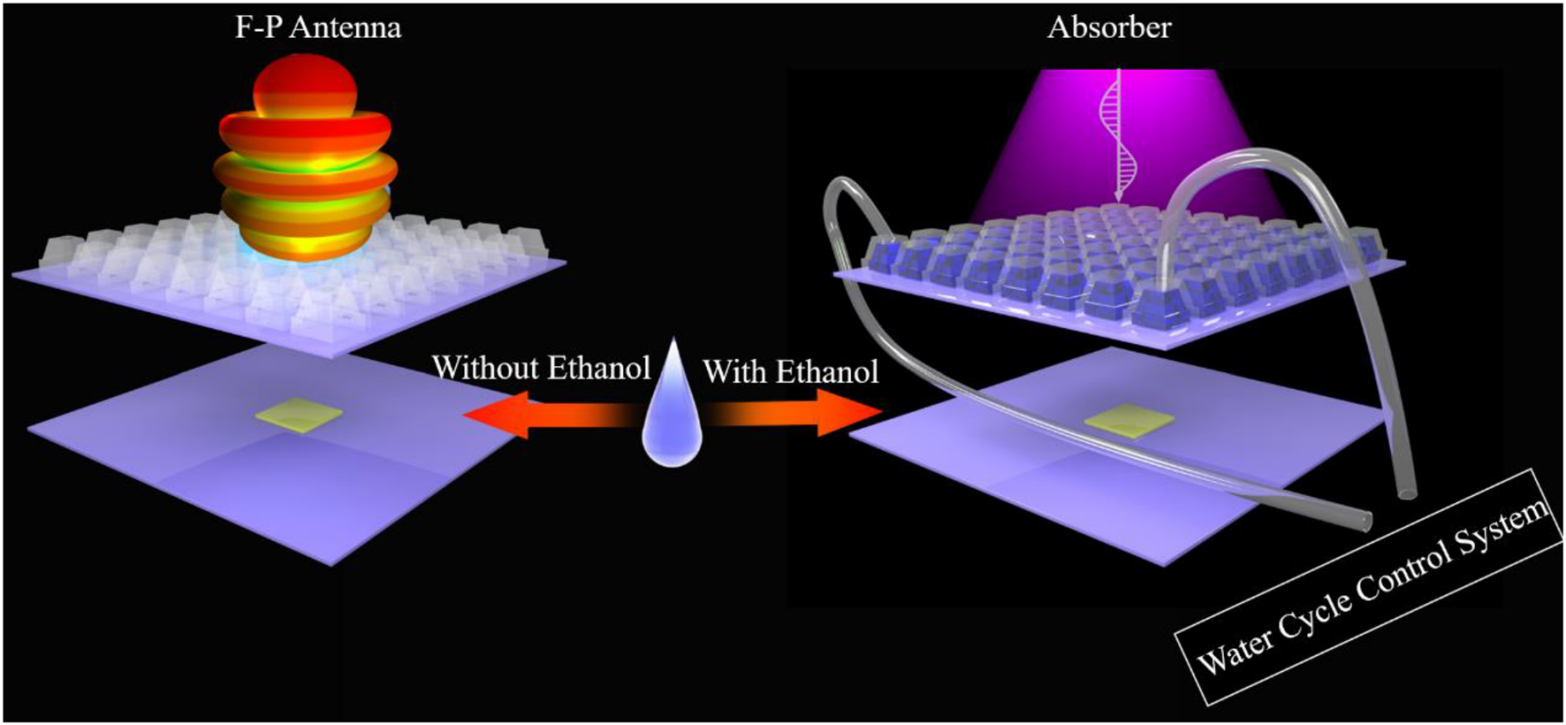
The working mechanism of the integrated structure.

The working mechanism of the integrated structure is shown in Fig. 1. The structure has two functions. One is the liquid absorber, the other is the FP antenna. When the ethanol solution is injected into the structure, the source antenna doesn’t work. Then the whole structure can be seen as the 3D container layer, the ethanol layer and the PRS. The PRS can be considered as the metal ground of the liquid absorber. In this way, the whole structure forms a wideband liquid absorber. On the contrary, when the ethanol solution is extracted from the structure, the source antenna comes into play. Then the whole system can be seen as the 3D container layer, the metal layer and the source. In this situation, the 3D container and the metal layer collaboratively form the PRS. It is noted that the amplitude and phase response of the PRS with 3D container is slightly different from that without 3D container. However, this slight difference can be overcome by changing the height of the FP resonator. The ethanol solution is controlled by the water cycle control system. The system used in this paper is the same as that in^17^. It takes about 40 s for the liquid to be completely evacuated from being filled. In this way, this structure can be used to switch between detection and stealth mode which can better adapt to the complex electromagnetic environment.

### The permittivity of the pure water and 45% ethanol solution

**Figure 2:**
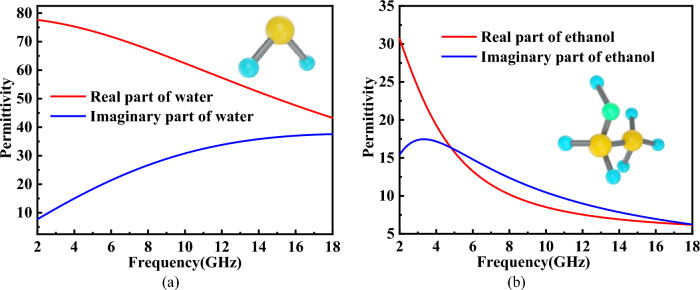
The permittivity of (**a**) pure water and (**b**) 45% ethanol solution.

The permittivity of pure water and 45% ethanol solution can both be described by the Debye formula. For pure water, the permittivity can be expressed by the formula as follows^31^:

1 $$\varepsilon (\omega ,{\rm T}) = \varepsilon_{\infty } ({\rm T}){ + }\frac{{\varepsilon_{0} ({\rm T}) - \varepsilon_{\infty } ({\rm T})}}{{1 - i\omega \tau ({\rm T})}}$$

where* ε*_∞_(*T*), *ε*_0_(*T*) and *τ*(*T*) are the optical permittivity, static permittivity, and rotational relaxation time, respectively. The *ε*_∞_(*T*), *ε*_0_(*T*) and *τ*(*T*) are all directly dependent on the temperature T:

2 $$\varepsilon_{0} \left( T \right) = a_{1} - b_{1} T{ + }c_{1} T^{2} - d_{1} T^{3}$$

3 $$\varepsilon_{\infty } \left( T \right) = \varepsilon_{0} \left( T \right) - a_{2} e^{{ - b_{2} T}}$$

4 $$\tau \left( T \right) = c_{2} e^{{\frac{{d_{2} }}{{T + T_{0} }}}}$$

where *a*_1_ = 87.9,* b*_1_ = 0.404 °C^−1^, *c*_1_ = 9.59 × 10^−4^ °C^−2^, *d*_1_ = 1.33 × 10^−6^ °C^−3^, *a*_2_ = 80.7, *b*_2_ = 4.42 × 10^−3^ °C^−1^, *c*_2_ = 1.37 × 10^−13^ s, *d*_2_ = 651 °C, *T*_0_ = 133 °C, *T* is the water temperature in °C. In our design, *T* is set as 25 °C which is close to room temperature. Therefore, *ε*_∞_(*T*), ε_0_(*T*) and *τ*(*T*) are constant. The permittivity of the pure water at 25 °C is shown Fig. 2a. Similarly, the permittivity of the 30% ethanol solution can also be described by formulas (1), (2), (3) and (4). The only difference is the values of *ε*_∞_(*T*),* ε*_0_(*T*) and τ(*T*). The values of *ε*_∞_(*T*), *ε*_0_(*T*) and *τ*(*T*) are respectively 5.0344, 735.1702 and 20.9273 which has been proposed in^30^. The permittivity of the 45% ethanol solution at 25 °C is shown Fig. 2b. From Fig. 2a and b, we can see that the imaginary part of pure water and 45% ethanol solution is very big. This means that these two materials have a large loss for electromagnetic waves and this can help us design a wideband liquid absorber. However, the real and imaginary parts of the permittivity of the 30% ethanol solution is smaller than the pure water. Considering the fluidity of the liquid layer and the good impedance matching, 45% ethanol is selected. The permittivity of the pure water and 45% ethanol solution will be used in analysis and simulation of the liquid absorber.

### Design of the FP antenna and the liquid absorber

**Figure 3:**
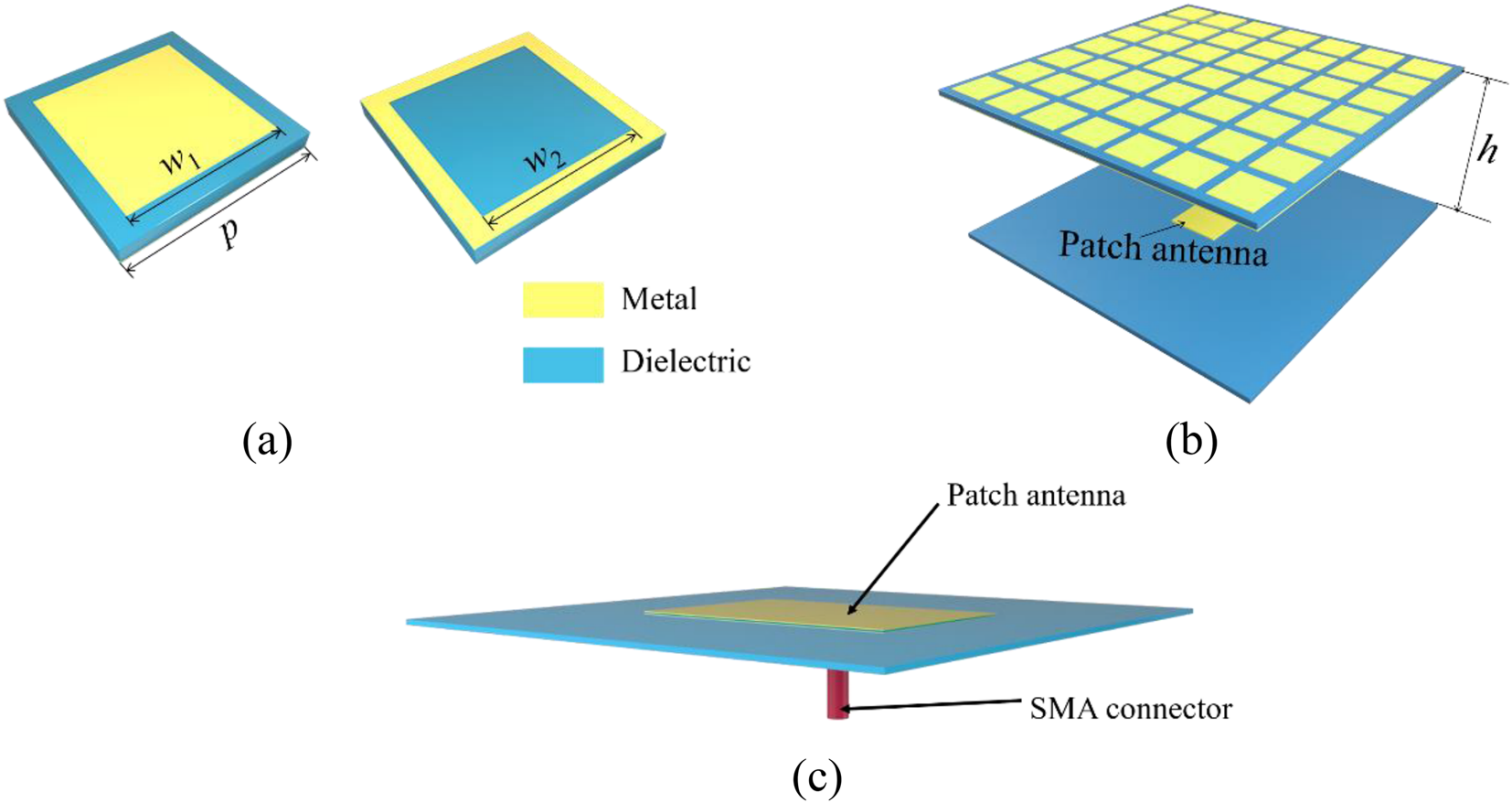
(**a**) Unit cell of the PRS (**b**) 3D structure of the FP antenna.(**c**) The feeding diagram of the source antenna.

The unit cell of the PRS is shown in Fig. 3a, it is composed of the top metal patch, the F4B dielectric substrate (*ε*_*r*_ = 2.2, tan*δ* = 0.0009) and the bottom metal loop. The PRS has two functions: (1) acts as the metal ground of the absorber; (2) forms an FP cavity with the source antenna floor. The FP antenna consists of the PRS and the patch antenna which is served as a source, as shown in Fig. 3b. The source antenna is fed by a SMA connector at the back of the patch as shown in Fig. 3c.

**Figure 4:**
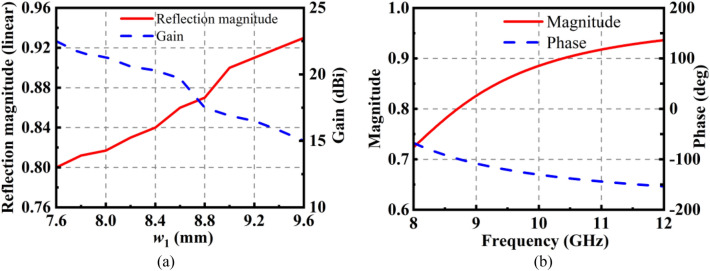
(**a**) Reflection magnitude of the PRS and the gain of the antenna with different *w*_1_ at 10 GHz. (**b**) Reflection magnitude and phase of the PRS.

Although it can improve the absorption property of the absorber with a high reflection magnitude of the PRS, the antenna’s gain will decrease. Therefore, it must make a compromise between the absorption property of the liquid absorber and the gain of the FP antenna. As shown in Fig. 4a, with the increase of the width of the top patch *w*_1_, the reflection magnitude of the PRS at 10 GHz will increase. However, the gain of the antenna at 10 GHz decreases as proposed forward. Last, the intersection of two curves of the reflection magnitude of PRS and the gain of the antenna has been chosen.

The height of the FP resonant cavity can be designed according to the following equation^1,33^:

5 $$h = \frac{\lambda }{4\pi }(\varphi_{1} - \varphi_{2} - 2N\pi ),N = 0,1,2...$$

where *φ*_1_ and *φ*_2_ are the reflection phase of PRS and the metal ground, respectively. *λ* is the wavelength of operating frequency. *N* is the resonance mode number of the resonant cavity and here only the zero-order (*N* = 0) is considered to make the antenna keep a low profile. Here, *φ*_1_ = − 130°, as shown in Fig. 4b. *φ*_2_ = 180° which is the reflection phase of the metal ground. *λ* is the wavelength of 10 GHz which is the operating frequency of the FP antenna in this work*.* Therefore, by Eq. (1), the calculated value of the height of the FP resonant cavity h = 9.6 mm. The optimized parameters are as follows: *w*_1_ = 8.6 mm, *p* = 12 mm, *w*_2_ = 8.6 mm.

**Figure 5:**
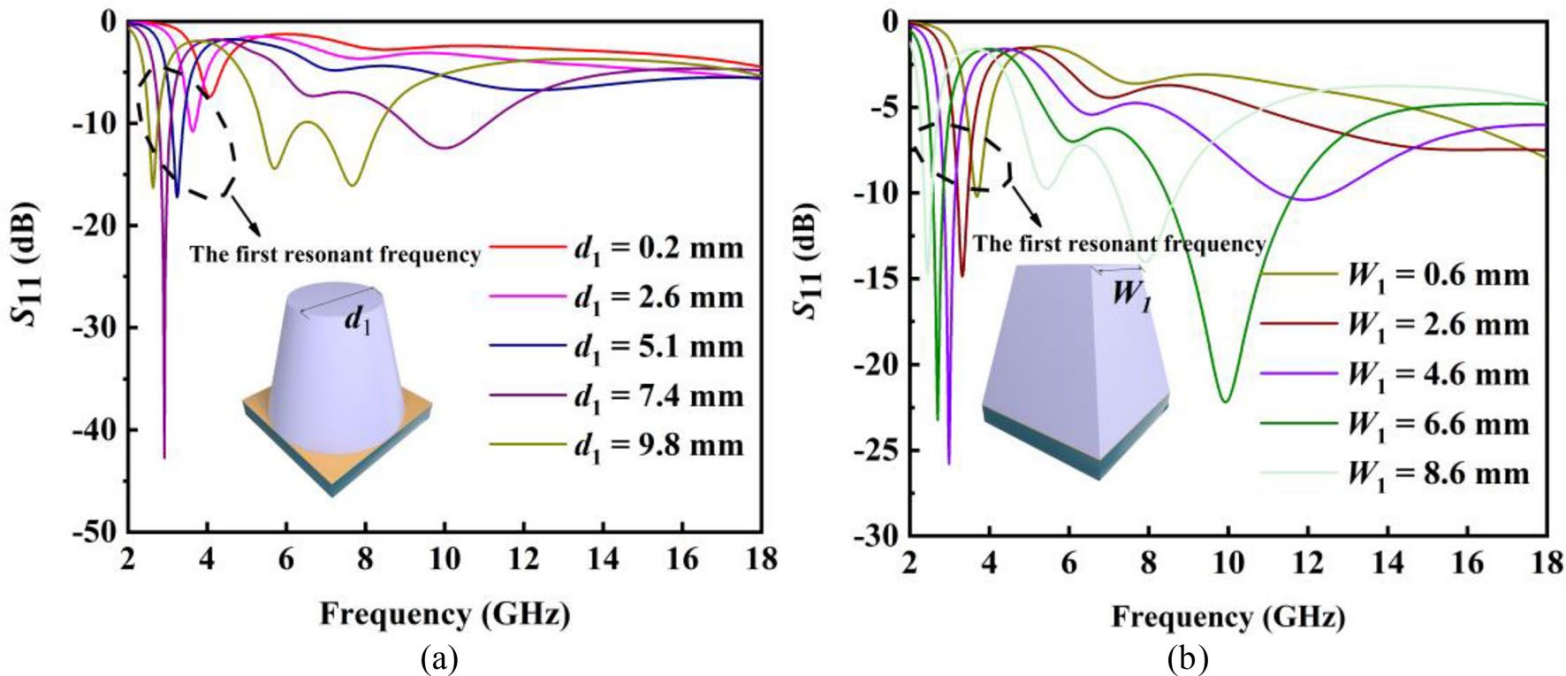
(**a**) *S*_11_ of the truncated cone-shaped liquid absorber with different top diameters *d*_1_ (**b**) *S*_11_ of the prismatic-shaped liquid absorber with different widths of top bottom *W*_1_.

As the finish of the PRS, the metal ground of the liquid absorber has been fixed. To expand the low-frequency absorption bandwidth of the liquid absorber under the fixed metal ground, two shapes of liquid absorbers are compared. One is the truncated cone-shaped structure, the other is the prismatic-shaped structure. Each unit cell of the liquid absorber consists of three layers: the 3d container layer (*ε*_*r*_ = 2.8, tan*δ* = 0.0318), the liquid layer and the metal ground. The only difference is their shape. As shown in Fig. 5a, keep the bottom diameter and the height of the truncated absorber unchanged, the first resonant frequency of the truncated cone-shaped absorber will go lower frequency with the increase of the top diameter *d*_1_. Similarly, the first resonant frequency of the prismatic absorber will also go lower frequency with the increase of the top width *W*_1_, as shown in Fig. 5b. However, compared to the truncated absorber, the first resonant frequency of the prismatic absorber is lower under a similar size.

To verify this conclusion, the Mie resonance theory has been introduced. According to^34^, the first resonant frequency can be described by the following formulas:

6 $$f = \frac{\varphi c}{{2\pi r\sqrt {\varepsilon_{p} \mu_{p} } }}$$

7 $$F(\varphi ) = \frac{2(\sin \varphi - \varphi \cos \varphi )}{{(\varphi^{2} - 1)\sin \varphi + \varphi cos\varphi }}$$

8 $$r = k_{0} \times \left( \frac{4}{3} \right)^{{k_{1} (h - n)}}$$

where *f* is the first-order Mie resonance, *r* is the radius of the spherical medium. c is the velocity of light, *ε*_*p*_ and *μ*_p_ are the permittivity and permeability of the material of the particle, respectively. *F*(*φ*) is the function which is related to *φ*. Under the first-order Mie resonance, *φ* = *π*. In^31^, a modified formula (8) has been given in the case of the water block. However, this doesn’t apply to our situation because there are more parameters of the liquid absorber which are analyzed. Therefore, formula (8) has been modified by the following:

9 $$r = k_{0} \times \left( \frac{4}{3} \right)^{{\left( {\sum\limits_{i = 1}^{n} {k_{i} } f_{i} } \right) + b}}$$

where *f*_*i*_ is the impact factor of the absorber. *n* is the number of the impact factor and *k*_*i*_ is the influence coefficient of each impact factor. b is the constant variable and is related to the shape of the liquid absorber.

**Figure 6:**
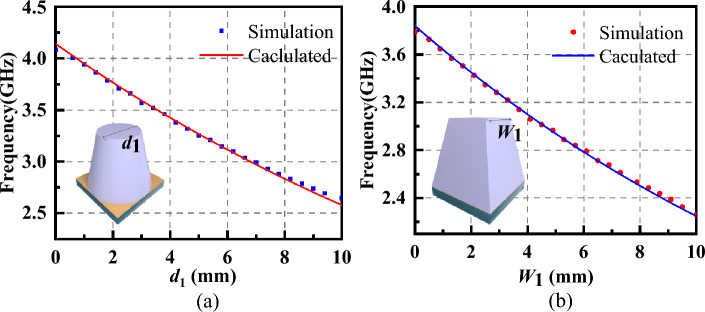
(**a**) The first resonant frequency of the truncated cone-shaped absorber with different top diameters *d*_1_. (**b**) The first resonant frequency of the prismatic-shaped absorber with different top widths *W*_1_.

Taking the truncated cone-shaped absorber as an example, three impact values have been considered: the basal diameter, the height of the absorber and the top diameter. Therefore, *r* can be calculated by the following equation:

10 $$r_{1} = k_{0} \times \left( \frac{4}{3} \right)^{{(k_{11} \times d_{1} + k_{12} \times d_{2} + k_{13} \times h_{11} ) + b_{1} }}$$

where *d*_1_, *d*_2_ and *h*_11_ are the top diameters, the bottom diameter and the height of the truncated cone-shaped absorber, respectively. Here, *k*_0_ = 0.005, *k*_11_ = 199.1, *k*_12_ = 164.8, *k*_13_ = 310.1, *b*_1_ = 9.58. Similarly, for the prismatic-shaped absorber, *r* can be described as:

11 $$r_{2} = k_{0} \times \left( \frac{4}{3} \right)^{{(k_{21} \times W_{1} + k_{22} \times W_{2} + k_{23} \times h_{21} ) + b_{2} }}$$

where *W*_1_, *W*_2_ and *h*_21_ is the top width, the bottom width and the height of the prismatic-shaped absorber, respectively. Here, *k*_21_ = 265.4, *k*_22_ = 186.1, *k*_23_ = 311.3, *b*_2_ = 9.81. To verify the correction of Eq. (4), The first resonant frequency of the truncated absorber with different top diameter *d*_1_ and the first resonant frequency of the prismatic absorber with different top width *W*_1_ have been shown in Fig. 6a and b, respectively.

From Eq. (6), we can find that *f* becomes smaller when *r* becomes bigger. When keeping the bottom diameter and the height of the truncated cone-shaped absorber unchanged, it can be known from Eq. (9) that with the increase of the top diameter,* r* will become larger, resulting in a decrease in *f*. This is consistent with the conclusion put forward. From Eqs. (10) and (11), we can find that *k*_21_ > *k*_11_, *k*_22_ > *k*_12_, *k*_23_ > *k*_13_ and *b*_1 _≈ *b*_2_. Therefore, when the two shaped absorbers have the same height (*h*_11_ = *h*_21_), metal ground (*d*_2_ = *W*_2_), and top length (*d*_1_ = *W*_1_), *r*_2_ > *r*_1_. In other words, the first frequency of the prismatic-shaped absorber is lower than that of the truncated cone-shaped absorber. Last, the prismatic absorber has been chosen to realize good absorption properties at low frequencies.

**Figure 7:**
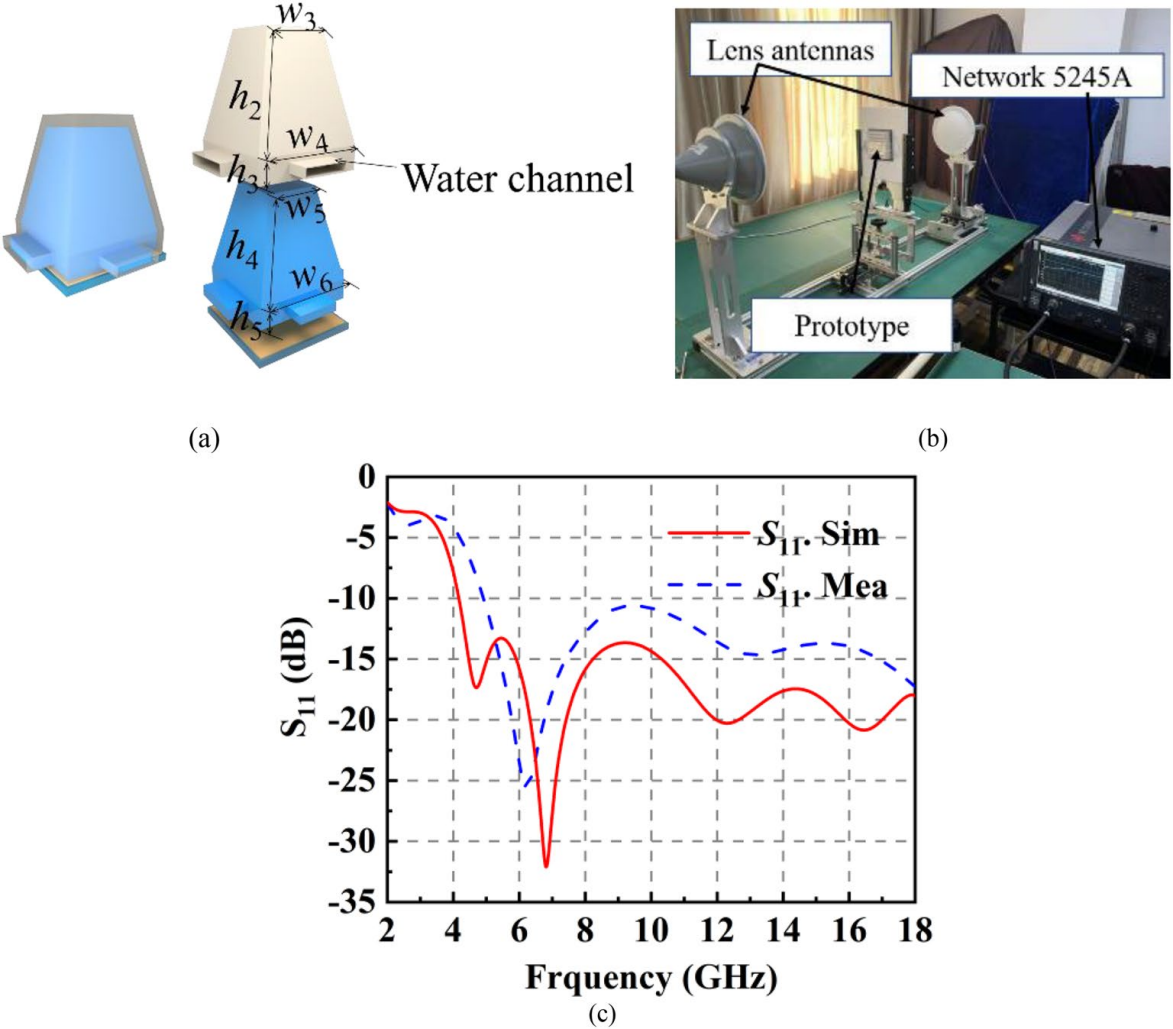
(**a**) The unit cell of the liquid absorber. (**b**) Measurement setup of the *S*_11_ of the absorber. (**c**) Simulated and measured *S*_11_ of the liquid absorber.

In order to facilitate processing, a base layer is added to the container and this almost has no effect on the property of the liquid absorber. The unit cell of the absorber has been shown in Fig. 7a. It is composed of three layers: the 3D container, the 45% ethanol layer and the metal. A prismatic 3D container has been designed to enclose the liquid. To facilitate the circulation of liquid between adjacent unit cells, the water channels have been designed between adjacent units. To achieve good impedance matching in a wide band, 45% ethanol is chosen to replace water. The reason has been introduced in^30^. The optimized parameters are shown as follows: *w*_3_ = 5 mm, *w*_4_ = 8 mm, *w*_5_ = 3 mm, *w*_6_ = 7 mm, *h*_2_ = 10 mm, *h*_3_ = *h*_5_ = 5 mm, *h*_4_ = 9 mm.

**Figure 8:**
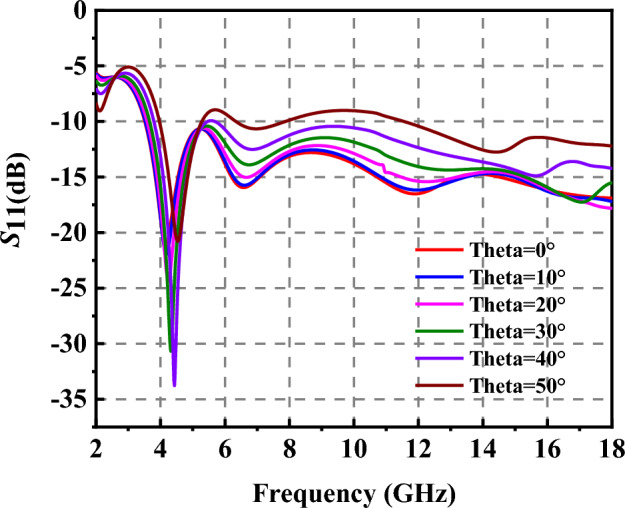
*S*_11_ of the prismatic-shaped liquid absorber with different incident angle.

The *S*_11_ of the absorber has been simulated and measured, as shown in Fig. 7c. It is noted that lens antennas^35^ and vector network analyzer 5245A are chosen to measure the *S*_11_ of the liquid absorber due to size constraints of the liquid absorber, as shown in Fig. 7b. The absorption band of the absorber ranges from 4 to 18 GHz. The difference between measurement and experiment is affected by the following factors: (1) it is difficult to ensure that the container is completely filled with ethanol; (2) a small amount of misalignment of the 3D container and ethanol concentration is unavoidable in such a setup. The absorption property of the liquid absorber can maintain until 50°, as shown in Fig. 8.

### Structure and the property of the integrated structure

**Figure 9:**
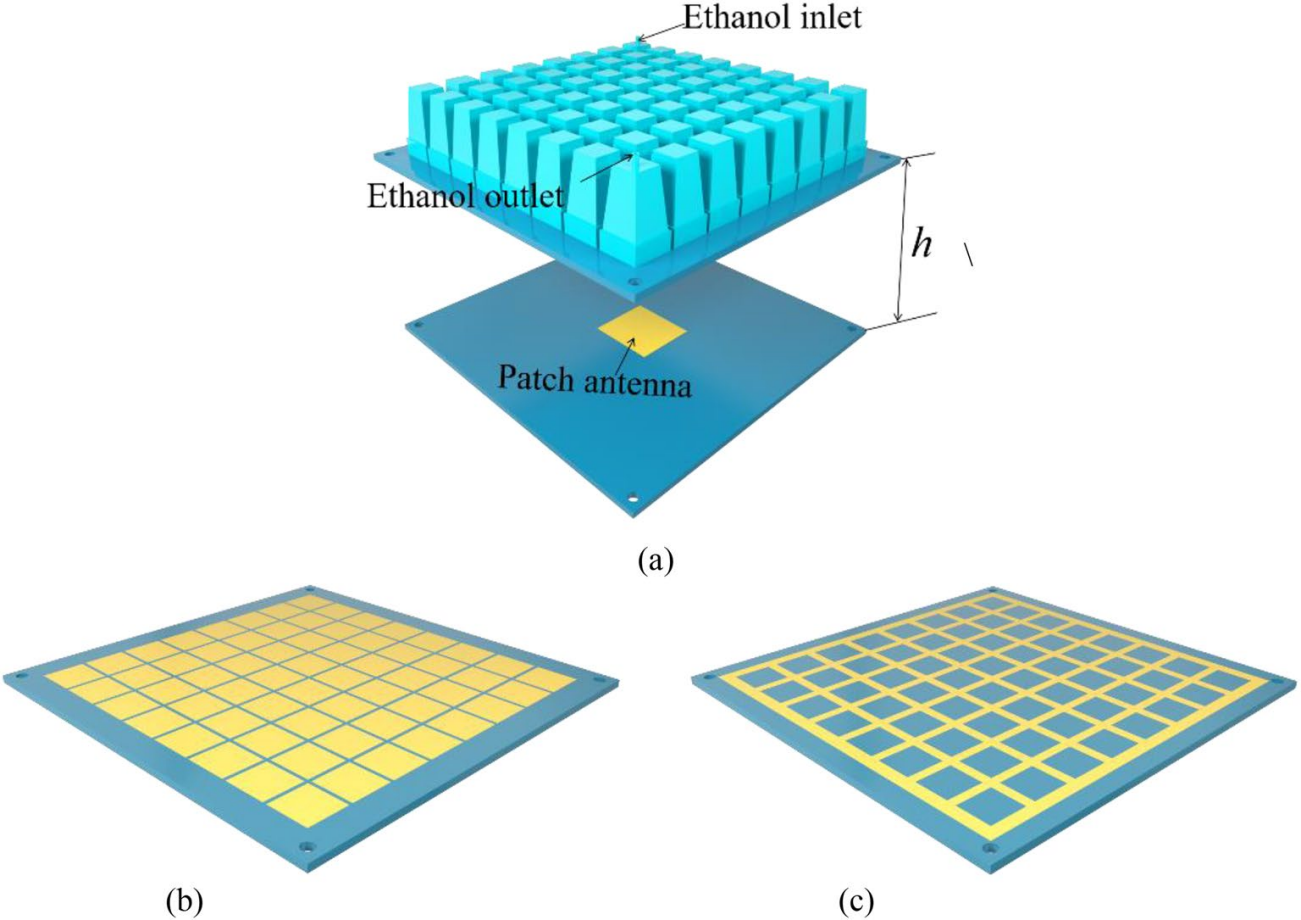
(**a**) 3D sketch of the FP antenna. (**b**) Front of the PRS. (**c**) Back of the PRS.

Figure 9a shows the 3D structure of the FP antenna. It is composed of a 3D container which is used to encapsulate ethanol. Channels are set up between each adjacent unit cell of the liquid absorber to ensure the circulation of the ethanol. An ethanol outlet and an ethanol inlet are designed to ensure fluid injection and extraction. Square patches and square loops are placed on both sides of the substrate which form the PRS, as shown in Fig. 9b and c. The patch antenna is used as the excitation source and it is fed by a SMA connector as proposed forward. It is noted that the height of the FP resonant cavity *h* with container is lower than that without container because of the existence of the 3D container.

**Figure 10:**
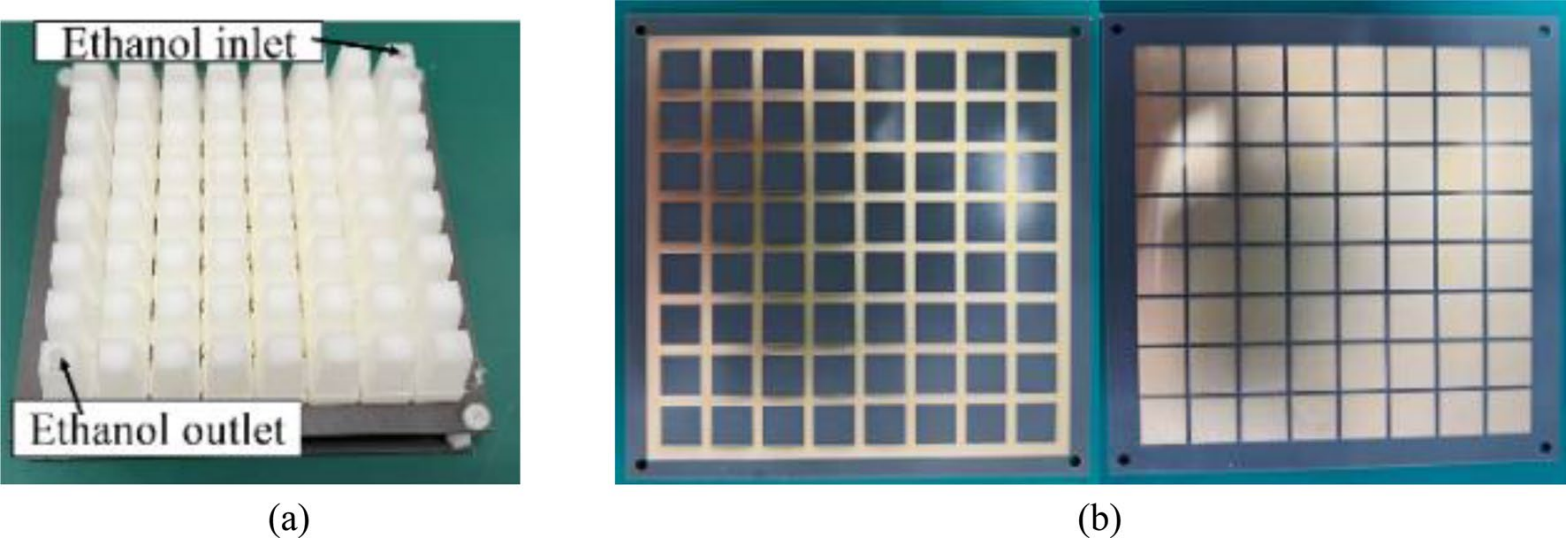
Photographs of the fabricated (**a**) FP antenna and (**b**) PRS.

To verify the performance of the FP antenna, the antenna has been processed and measured. The container structure which is used to enclose liquid is fabricated by 3D printed technology. The PRS and the microstrip patch antenna are fabricated by the PCB technology. The container is installed on the PCB board with glass glue to ensure that the entire container is watertight. Figure 10a and b show photographs of the fabricated FP antenna and PRS.

**Figure 11:**
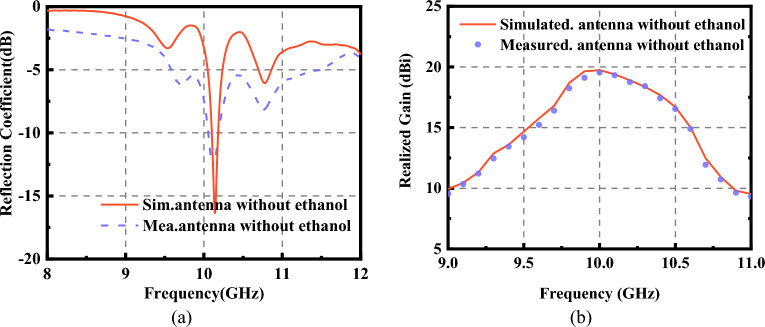
Simulated and measured (**a**) *S*_11_ and (**b**) Realized gain of the FP antenna.

**Figure 12:**
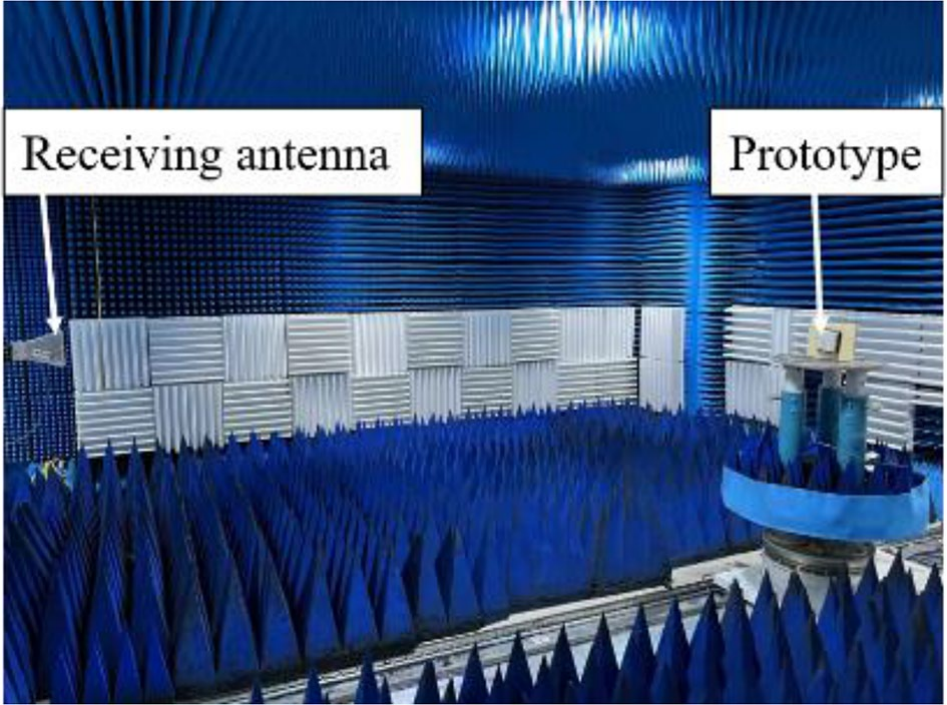
Measurement setup of the radiation performance.

**Figure 13:**
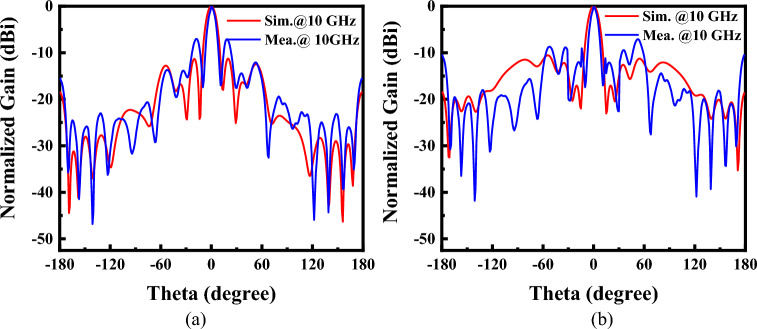
Radiation patterns of the proposed antenna at 10 GHz. (**a**) E-plane (**b**) H-plane.

**Figure 14:**
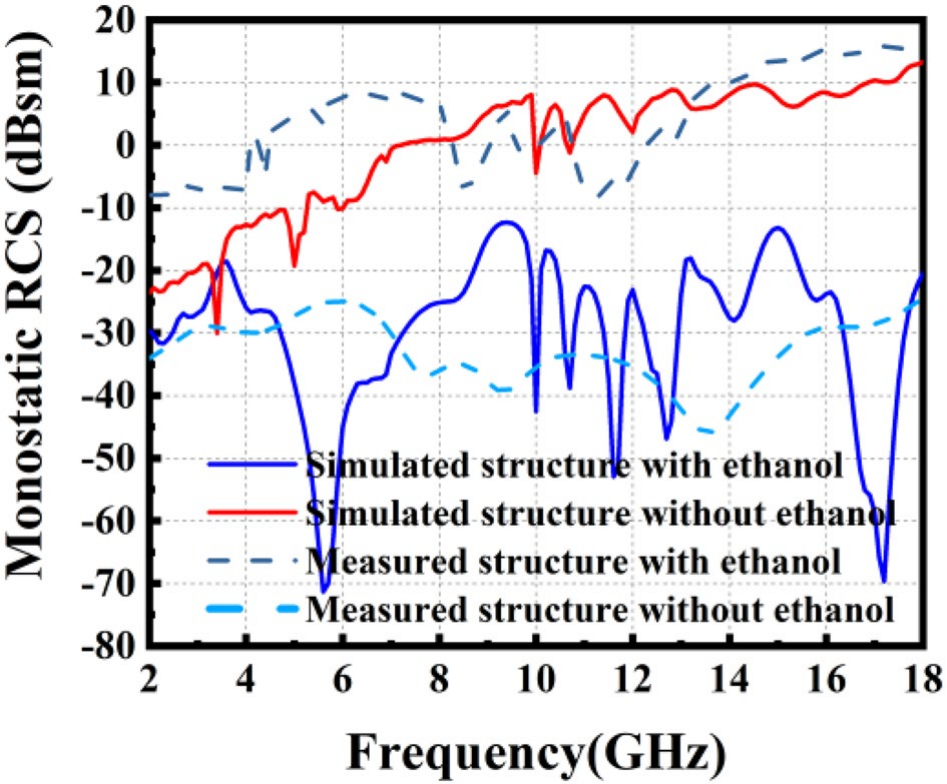
Monostatic RCS of the integrated structure antenna for normal incidence.

The simulated and measured *S*_11_ of the FP antenna is shown in Fig. 11a. The operating frequency is about 10 GHz. The operating bandwidth is about 100 MHz. The antenna gain at 10 GHz is 19.7 dB, as shown in Fig. 11b. The radiation pattern of the FP antenna has been measured. The test scene is shown in Fig. 12. The radiation patterns of the antenna have been shown in Fig. 13a and b. The monostatic RCS of the structure is shown in Fig. 14. We can find that the band of RCS reduction is consistent with the absorption band of the absorber. When the frequency is below 4 GHz, the RCS of the two states of the structure keeps almost unchanged. When the frequency is higher than 4 GHz in the absorption band of the absorber, the RCS reduction is noticeable. The average RCS reduction is 28.4 dBsm. To better explain the good properties of the proposed structure, a comparison between the existing low-RCS antennas or metasurfaces has been made. As shown in Table 1, it can be found that this structure has a wideband RCS reconfigurable band and wide absorption band. The gain of the antenna has been significantly increased compared to the antennas which is designed by liquid materials.

**Table 1:** Comparisons (Simulated Results) with other structures.

References	Metasurface/antenna	Reconfigurable RCS	Reconfigurable bandwidth of RCS	Bandwidth of RCS reduction (%)	Gain
^14^	Antenna	No	–	100	8 dB
^15^	Antenna	No	–	80	12 dB
^16^	Anetenna	No	–	52	20 dB
^20^	Antenna	No	–	96	6 dB
^36^	Metasurface	Yes	32%	32	–
^21^	Antenna	Yes	40%	40	6 dB
^23^	Antenna	Yes	24%	24	10 dB
^30^	Antenna	Yes	160%	160	12 dB
This work	Antenna	Yes	133%	116	19.7 dB

## Conclusion

A structure that can serve as the FP antenna or wideband liquid absorber has been proposed in this paper. When the ethanol is injected, the structure acts like an absorber. When the ethanol is extracted, the structure acts like an FP antenna. This can be applied to the radar system stealth. With the convertible modes, the structure can adapt the complex electromagnetic environment. When the ethanol is injected to the structure, the structure acts as the liquid absorber which contributes to the stealth of the radar system. The absorption band ranges from 4 to 18 GHz. The absorption bandwidth is over 133%. When ethanol is extracted from the structure, the structure acts as the FP antenna. The antenna operates at 10 GHz and the gain of the antenna is 19.7 dBi which can help the radar system detect. The structure possesses reconfigurable RCS by injecting or extracting and this can adapt to the complex electromagnetic environment. The RCS of the integrated structure is reduced from 4 to 18 GHz and 28.4 dB average RCS reduction is realized. The formula of the Mie resonance theory has been extended to a different structure of liquid absorber and this can help instruct readers to design liquid absorbers.

## Data Availability

The dataset used and/or analyzed during the current study will be made available by the corresponding author on reasonable request.

## References

[1] Feresidis AP, Goussetis G, Wang S, Vardaxoglou JC (2005). Artificial magnetic conductor surfaces and their application to low-profile high-gain planar antennas. IEEE Trans. Antennas Propag..

[2] Konstantinidis K, Feresidis AP, Hall PS (2014). Multilayer partially reflective surfaces for broadband Fabry–Perot cavity antennas. IEEE Trans. Antennas Propag..

[3] Gardelli R, Albani M, Capolino F (2006). Array thinning by using antennas in a Fabry–Perot cavity for gain enhancement. IEEE Trans. Antennas Propag..

[4] Hashmi RM, Zeb BA, Esselle KP (2014). Wideband high-gain EBG resonator antennas with small footprints and all-dielectric superstructures. IEEE Trans. Antennas Propag..

[5] Pan W, Huang C, Chen P, Ma X, Hu C, Luo X (2014). A low-RCS and high-gain partially reflecting surface antenna. IEEE Trans. Antennas Propag..

[6] Li W, Cao X, Gao J (2016). Broadband RCS reduction and gain enhancement microstrip antenna using shared aperture artificial composite material based on quasi-fractal tree. IET Microw. Antennas Propag..

[7] Mu J, Wang H, Wang H, Huang Y (2017). Low-RCS and gain enhancement design of a novel partially reflecting and absorbing surface antenna. IEEE Antennas Wirel. Propag. Lett..

[8] Jiang H, Xue Z, Li W, Ren W, Cao M (2016). Low-RCS high-gain partially reflecting surface antenna with metamaterial ground plane. IEEE Trans. Antennas Propag..

[9] Zheng Y (2018). Wideband gain enhancement and RCS reduction of Fabry–Perot resonator antenna with chessboard arranged metamaterial superstrate. IEEE Trans. Antennas Propag..

[10] Mol VL, Aanandan CK (2018). Radar cross section reduction of low profile Fabry–Perot resonator antenna using checker board artificial magnetic conductor. Adv Electromagn..

[11] Zarbakhsh S, Akbari M, Samadi F, Sebak A (2019). Broadband and high-gain circularly-polarized antenna with low RCS. IEEE Trans. Antennas Propag..

[12] Liu Z, Liu S, Zhao X, Kong X, Huang Z, Bian B (2020). Wideband gain enhancement and RCS reduction of Fabry–Perot antenna using hybrid reflection method. IEEE Trans. Antennas Propag..

[13] Li K, Liu Y, Jia Y, Guo YJ (2017). A circularly polarized high-gain antenna with low RCS over a wideband using chessboard polarization conversion metasurfaces. IEEE Trans. Antennas Propag..

[14] Long M, Jiang W, Gong S (2017). Wideband RCS reduction using polarization conversion metasurface and partially reflecting surface. IEEE Antennas Wirel. Propag. Lett..

[15] Liu Z (2020). A low-RCS, high-GBP Fabry–Perot antenna with embedded chessboard polarization conversion metasurface. IEEE Access.

[16] Zhang L (2017). Realization of low scattering for a high-gain Fabry–Perot antenna using coding metasurface. IEEE Trans. Antennas Propag..

[17] Alibakhshikenari M, Virdee BS, Althuwayb AA (2021). Study on on-chip antenna design based on metamaterial-inspired and substrate-integrated waveguide properties for millimetre-wave and THz integrated-circuit applications. J. Infrared Millim. Terahertz Waves.

[18] Alibakhshikenari M, Virdee BS, See CH (2020). High-gain metasurface in polyimide on-chip antenna based on CRLH-TL for sub-terahertz integrated circuits. Sci. Rep..

[19] Alibakhshikenari M, Virdee BS, See CH (2020). Study on improvement of the performance parameters of a novel 0.41–0.47 THz on-chip antenna based on metasurface concept realized on 50 μm GaAs-layer. Sci. Rep..

[20] Liu P, Li Y, Zhang Z (2019). Circularly polarized 2 bit reconfigurable beam-steering antenna array. IEEE Trans. Antennas Propag..

[21] Liu Y, Zhang W, Jia Y, Wu A (2021). Low RCS antenna array with reconfigurable scattering patterns based on digital antenna units. IEEE Trans. Antennas Propag..

[22] Gao W, Chen SJ, Withayachumnankul W, Fumeaux C (2019). Horizontally polarized 360° beam-steerable frequency-reconfigurable antenna. IEEE Trans. Antennas Propag..

[23] Zhang J, Liu Y, Jia Y, Zhang R (2021). High-gain Fabry–Pérot antenna with reconfigurable scattering patterns based on varactor diodes. IEEE Trans. Antennas Propag..

[24] Ouedraogo RO, Rothwell EJ, Greetis BJ (2011). A reconfigurable microstrip leaky-wave antenna with a broadly steerable beam. IEEE Trans. Antennas Propag..

[25] Andryieuski A, Kuznetsova SM, Zhukovsky SV, Kivshar YS, Lavrinenko AV (2015). Water: Promising opportunities for tunable all-dielectric electromagnetic metamaterials. Sci. Rep..

[26] Yoo YJ, Ju S, Park SY, Ju Kim Y, Bong J, Lim T, Kim KW, Rhee JY, Lee YP (2015). Metamaterial absorber for electromagnetic waves in periodic water droplets. Sci. Rep..

[27] Shrekenhamer D, Chen WC, Padilla WJ (2013). Liquid crystal tunable metamaterial absorber. Phys. Rev. Lett..

[28] Todorov TK, Reuter KB, Mitzi DB (2010). High-efficiency solar cell with earth-abundant liquid-processed absorber. Adv. Mater..

[29] Hu Z, Wang S, Shen Z, Wu W (2017). Broadband polarization-reconfigurable water spiral antenna of low profile. IEEE Antennas Wirel. Propag. Lett..

[30] Zou Y (2022). A slot antenna array with reconfigurable RCS using liquid absorber. IEEE Trans. Antennas Propag..

[31] Yan X (2020). Water-based reconfigurable frequency selective rasorber with thermally tunable absorption band. IEEE Trans. Antennas Propag..

[32] Kong X, Kong L, Jiang S, Wang X, Zou Y, Xing L (2021). Low-profile and dual-polarization water-based frequency selective rasorber with ultrawideband absorption. IEEE Antennas Wirel. Propag. Lett..

[33] Trentini GV (1956). Partially reflecting sheet arrays. IEEE Trans. Antennas Propag..

[34] Lewin L (1947). The electrical constants of a material loaded with spherical particles. J. Inst. Electr. Eng. Part III.

[35] Ghodgaonkar DK, Varadan VV, Varadan VK (1989). A free-space method for measurement of dielectric constants and loss tangents at microwave frequencies. IEEE Trans. Instrum. Meas..

[36] Luo X-Y (2020). Active cylindrical metasurface with spatial reconfigurability for tunable backward scattering reduction. IEEE Trans. Antennas Propag..

